# Enhanced Neurogenesis in the Hippocampal Dentate Gyrus during Antigen-Induced Arthritis in Adult Rat – A Crucial Role of Immunization

**DOI:** 10.1371/journal.pone.0089258

**Published:** 2014-02-21

**Authors:** Johannes Leuchtweis, Michael K. Boettger, Fanny Niv, Christoph Redecker, Hans-Georg Schaible

**Affiliations:** 1 Institute of Physiology 1/Neurophysiology, Jena University Hospital – Friedrich Schiller University of Jena, Jena, Germany; 2 Hans Berger Department of Neurology, Jena University Hospital – Friedrich Schiller University of Jena, Jena, Germany; University of Arizona, United States of America

## Abstract

Neurogenesis in the subgranular zone of the mammalian hippocampal dentate gyrus contributes significantly to brain neuroplasticity. There is evidence that inflammation of the central nervous system inhibits neurogenesis but peripheral inflammation such as antigen-induced arthritis may rather enhance neurogenesis. Manifest arthritis is associated with symptoms such as pain and altered locomotion indicating that peripheral inflammation is associated with changes of both the immune system and the nervous system. This raises the intriguing question whether immune or neuronal factors or both actually drive changes of neurogenesis. Here we explored hippocampal neurogenesis in the rat during chronic antigen-induced arthritis in the knee joint. We analyzed neurogenesis in control rats, and in rats which were immunized for the antigen producing arthritis but which did not show arthritis and neurological symptoms, and in rats in which antigen injection into the knee produced manifest local inflammation and symptoms such as pain at the inflamed knee and altered locomotor behavior. Neurogenesis was assessed by quantifying bromodeoxyuridine-positive cells in sections of the complete hippocampal dentate gyrus. Compared to control animals, rats with antigen-induced arthritis presenting manifest local inflammation, hyperalgesia at the inflamed knee and significantly altered locomotion exhibited a significant increase of bromodeoxyuridine-positive cells. However, a similar increase in the number of such cells was found in rats which were only immunized against the antigen, but in which no local inflammatory response was induced and which thereby neither showed hyperalgesia nor alterations of locomotion. Thus we conclude that in peripheral immune-mediated arthritis the activation of the immune system in the process of immunization is the causal factor driving enhanced neurogenesis, and neither the local enhancement of inflammation nor the activation of the nervous system leading to neurological symptoms such as pain and altered locomotion. It seems noteworthy to further explore the clinical importance of this neuroimmune interaction.

## Introduction

Peripheral inflammation such as arthritis causes pain, guarding behavior, and other pain-related disturbances such as fatigue [1], [2]. These symptoms are generated by short- and long-term changes in the nervous system such as the sensitization of nociceptive pathways, the regulation of multiple pain-related ion channels, mediators and receptors, glial activation and others [2]–[5]. However, there is increasing evidence that painful diseases, in particular when they become chronic, not only affect the nociceptive system. They also affect brain functions which have an important role in the processes of cognition, learning, adaptation to altered environmental conditions, and changes of mood [6]. In addition, chronic arthritis is characterized by substantial neuroendocrine changes which affect inflammation [7]. In general the response pattern of the brain to peripheral inflammation and the neuroplastic changes resulting from peripheral inflammation are not well understood.

Neurogenesis in the subgranular zone (SGZ) of mammalian hippocampal dentate gyrus (DG) is an important mechanism of brain neuroplasticity. Adult neurogenesis whereat neural stem/progenitor cells (NSCs) proliferate into neuronal or glial progenitors [8] occurs throughout life in circumscribed brain areas [9], [10]. The newly generated neurons migrate into the granule cell layer of the DG and integrate into the existing hippocampal circuitry [11]. They modulate brain performance under altered environmental conditions, and the new cells may contribute to synaptic plasticity and are thought to be involved in long-term potentiation and depression (LTP/LTD) [12]–[15]. It was suggested that cognitive brain functions such as learning and memory involve adult neurogenesis in the hippocampus [16]. Adult neurogenesis in the SGZ can be up- or downregulated by a wide variety of factors such as aging [17], psychosocial [18], [19] and physical stress [20]–[22], irradiation [23], enriched environment [24], and physical exercise [25].

The dentate gyrus also responds to different types of pathophysiology with significant changes in neurogenesis. Adult neurogenesis may increase in the context of acute pathophysiological insults but this does not necessarily represent beneficial adaptations of the hippocampal network which promote reorganization and recovery. In the context of epilepsy and stroke, significant portions of newborn neurons form aberrant dendritic arborization and connectivity which impair hippocampal function [25]–[28]. Adult neurogenesis is also altered by proinflammatory cytokines. Some cytokines inhibit neurogenesis [29] whereas others stimulate it [30]. Several studies showed that inflammatory conditions, in particular within the central nervous system, inhibit neurogenesis [31]–[33]. By contrast, neurogenesis was enhanced in the experimental model of antigen-induced arthritis (AIA) in mice [34].

AIA is a suitable model of immune-mediated arthritis such as human rheumatoid arthritis because it shows typical features of rheumatoid arthritis such as a progression from an acute inflammatory to a chronic inflammatory/destructive state with flare-up reactions [35], [36]. Enhanced neurogenesis during AIA suggests, therefore, that not only inflammation in the nervous system but even peripheral inflammation has a significant impact on the proliferation of neural stem/progenitor cells (NSCs). However, immune-mediated arthritis is not only an inflammatory immune process. It has significant neurological consequences such as pain and considerable changes of gait indicating substantial neuronal activation and neuroplasticity [35], [36]. Because of this complexity it is difficult to attribute changes of neurogenesis either to changes in the immune system or to changes in the nervous system or both. In the present study we made an attempt to identify which factors cause enhanced neurogenesis. We investigated dentate neurogenesis in different groups of animals. We quantified neurogenesis (a) in control animals without immunization and inflammation, (b) in rats which were immunized only (in these rats the immune system is challenged but rats do usually not show neurological symptoms), and (c) in rats in which we induced AIA after immunization (these rats show manifest inflammation, pain and altered locomotion). We found that chronic unilateral inflammation in the knee joint is associated with enhanced neurogenesis. The main factor inducing neurogenesis seems to be the systemic reaction to immunization and neither manifest inflammation in the knee nor the symptoms pain and altered locomotion.

## Materials and Methods

This study was carried out in strict accordance with EC regulations for the care and use of laboratory animals. The protocol was approved by the Thuringian state authorities for animal protection (registration number 02-013/08). All injections for immunization and BrdU application, and induction of monoarticular AIA were performed under short anesthesia with 3% isoflurane.

### Antigen-Induced Arthritis (AIA)

**Figure 1:**
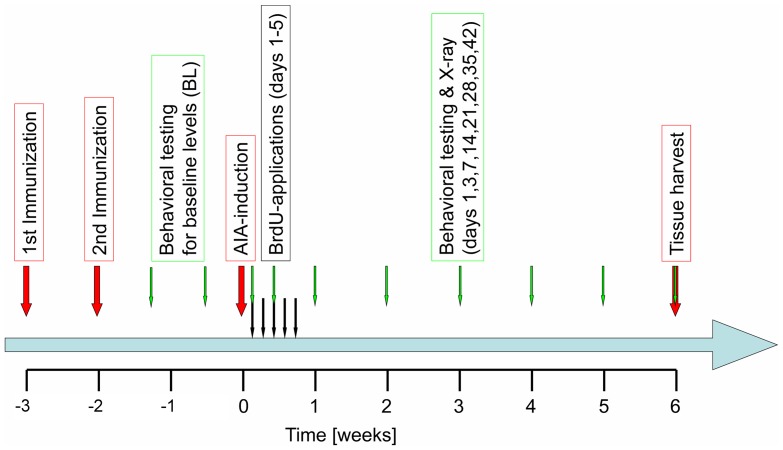
Experimental protocol of the study. BrdU - bromodeoxyuridine.

Thirty-four female Lewis rats (age 6 to 8 weeks upon arrival, weighing 160 to 180 g, Charles River, Sulzfeld, Germany) were used for the studies. Lewis rats are particularly susceptible to AIA [37] and also particularly suitable for behavioral experiments because they are quite tame. Animals were housed in groups of 4 animals per standard cage in a climate-controlled room on a 12∶12 h light:dark cycle with water and standard rodent chow available *ad libitum*. AIA was induced as reported previously [35], [38]. For immunization, 500 µg of antigen [methylated bovine serum albumin (mBSA), Sigma, Deisenhofen, Germany] in saline emulsified with 500 µL of Freund’s complete adjuvant (Sigma) supplemented with 2 mg/mL *Mycobacterium tuberculosis* strain H37RA (Difco, Detroit, MI, USA) were injected subcutaneously twice with a one-week interval between immunizations (Fig. 1). After another two weeks, a sterile mBSA solution (500 µg in 50 µL) was injected into the left knee joint cavity to induce monoarticular AIA in 7 rats. The experimental design of the whole experiment is shown in Fig. 1.

### Experimental Groups

Animals were divided randomly into 5 experimental groups. In 7 rats AIA was induced (AIA group). Another group of 10 animals were only immunized but did not get the intraarticular injection of mBSA [“immunized control” (IC) group]. Another 7 rats received no treatment [“naïve control” (NC) group], 7 rats underwent only X-ray exposure (CXray group), and 3 rats were only immunized but they were neither exposed to X-ray nor were they tested for changes in pain and locomotor behavior (IC-wt = “immunized control without testing” group). The IC-wt group consists of only 3 animals, because of tissue-damage after brain removal.

### Pain-related Behavior and Grading of Arthritis

Primary hyperalgesia at the inflamed knee was assessed with a dynamometer (Correx, Bern, Switzerland) [39]. Increasing pressure was applied to the lateral side of the knee joint at the level of the joint space until the animals attempted to escape or vocalized [35]. The applied weight force was read out in grams. A cutoff value of 250 g was defined to prevent tissue damage. Testing was performed once for each animal on each testing day to avoid nociceptive sensitization due to repeated testing (see Figure 1).

Weight bearing as a functional measure of pain-related guarding behavior of the inflamed hindlimb was assessed with an incapacitance tester (Linton Instrumentation, Norfolk, UK). For that purpose animals were placed with both hind paws on scales inside a plastic cage. The weight force resting on the two scales was consecutively measured for three times after animals acclimated and sat calm in the cage. From these averaged values, the relative weight (in %) resting on the inflamed hindlimb was evaluated [weight on inflamed hindlimb × 100%/(weight on inflamed hindlimb+weight on non-inflamed hindlimb)] as described previously [40]. The mediolateral diameter of each knee was assessed using a vernier calliper (Mitutoyo, Neuss, Germany) on every testing day for each animal. The relative swelling was calculated by subtracting the diameter of the non-inflamed knee from that of the inflamed one. All efforts were made to minimize suffering of the animals.

### Videoradiography

Videoradiographic analysis was performed as reported in detail previously [36] in the AIA and IC group. On each testing day, animals were allowed to walk spontaneously into a 2 m long plexiglass tunnel that was darkened at one end so that animals followed their instinct to go into the dark. Radiographs were obtained with a sampling rate of 500 Hz in a lateral perspective using a digital high-speed X-ray system (Neurostar; Siemens, Erlangen, Germany) in the middle part of the tunnel, when animals walked with constant speed. For each animal and each testing day, four runs were recorded, corresponding to approximately eight complete step cycles. Using defined landmarks, longitudinal axes of the femur and the tibia were calculated for each frame and the knee joint angle was calculated as the posterior angle between the two bone axes. The range of motion (ROM) in the knee joint was then obtained from the maximum and minimum joint angles for a complete step cycle. The radiation intensity of the X-ray system was 14.3 µSv/s. Animals of the CXray group were placed in a small plexiglass box directly in the radiation area for 2 s on each testing day without the possibility to walk.

### Immunohistochemical Staining of the Hippocampus

In order to identify proliferating cells all animals received daily intraperitoneal injections of bromodeoxyuridine (BrdU, 50 mg/kg; Sigma-Aldrich, Taufkirchen, Germany) on 5 consecutive days beginning on day 1 of the experimental time-course under short anesthesia with 3% isoflurane. On day 42, animals were deeply anesthetized with 120 mg/kg sodium thiopentone injected intraperitoneally (Trapanal; Byk Gulden, Konstanz, Germany) and transcardially perfused with 4% ice-cold phosphate-buffered paraformaldehyde (PFA) (see Figure 1). Whole brains were directly removed, postfixed for 24 hours in 4% PFA and equilibrated in 30% sucrose. Whole brains were cut on a freezing microtome in sequential 40 µm transversal sections and stored at −20°C in cryoprotectant. Immunocytochemistry for BrdU and immunofluorescent double labelling for BrdU and neuronal nuclei (NeuN) were performed similarly as described previously [41].

For immunoperoxidase staining, free-floating sections were rinsed in TBS (6 times, 15 min), incubated in 0.6% H_2_O_2_ (30 min at room temperature), rinsed in TBS (3 times, 15 min), treated with 2 N HCl for DNA denaturation (30 min at 37°C) and rinsed in 0.1 M boric acid (10 min at room temperature), then rinsed in TBS (3 times, 15 min), incubated in blocking solution (3% donkey serum and 0.1% Triton X-100 in TBS, 30 min at room temperature) followed by incubation with the primary antibody (monoclonal rat anti-BrdU IgG, 1∶500, AbD Serotec, Kidlington, UK) in blocking solution at 4°C overnight. After rinsing in TBS (3 times, 15 min), sections were incubated in blocking solution (30 min at room temperature) and incubated with the secondary antibody (Biotin-SP-conjugated donkey anti-rat IgG, 1∶500, Jackson Immuno Research, West Grove, PA, USA) for 2 hours at room temperature. After rinsing in TBS (3 times, 10 min) the slices were incubated in ABC solution (Vectastain Elite Kit, Vector Laboratories, Burlingame, CA, USA) for 1 hour at room temperature. After rinsing in TBS (3 times, 15 min) the slices were processed using diaminobenzidine hydrochloride (DAB, Sigma-Aldrich, Taufkirchen, Germany) as the chromogen, rinsed in TBS (6 times, 10 min) and finally mounted onto gelatine-coated slides, air-dried and coverslipped with Entellan (Merck, Darmstadt, Germany).

For double immunofluorescence staining the brain sections were pretreated in the same manner as described for immunoperoxidase staining omitting the H_2_O_2_-treatment. Sections were incubated with primary antibodies (monoclonal rat anti-BrdU IgG, 1∶500, AbD Serotec, Kidlington, UK and mouse anti-neuronal nuclei antigen (NeuN), 1∶500, Chemicon, Temecula, CA, USA) in blocking solution at 4°C overnight. After rinsing in TBS (3 times, 15 min), sections were incubated in blocking solution (30 min at room temperature) and incubated for 2 hours at room temperature with the secondary antibodies Rhodamine Red-X-conjugated AffiniPure Donkey Anti-Rat IgG (1∶200; Jackson Immuno Research, West Grove, PA, USA) and Alexa Fluor 488 donkey anti-mouse (1∶200; Invitrogen Molecular Probes, Eugene, Oregon, USA). Sections were finally rinsed in TBS (6 times, 10 min), mounted onto gelatine-coated slides, air-dried and coverslipped with Aqua-Poly/Mount (Polysciences, Eppelheim, Germany).

### Quantification of Cell Counts

Peroxidase staining was performed on every sixth section (in order to avoid counting of neurons twice) and the total number of BrdU-positive cells was counted in the complete dentate gyrus with an Axioplan 2 microscope (Zeiss, Jena, Germany). The number of BrdU-positive cells was multiplied by 6 to calculate the total number of BrdU-positive cells in the whole dentate gyrus as it was reported in previous studies [42], [43]. Immunofluorescent staining was performed to identify the neuronal phenotype of the BrdU-positive cells. Therefore 30 BrdU-positive cells were determined by colocalization of anti-BrdU IgG and NeuN using confocal laser scanning microscopy (Leica TCS SP5; Leica, Wetzlar, Germany) and the percentage of BrdU/NeuN-double-positive cells was calculated.

### Statistical Analysis

SPSS 19.0.0.1 software (SPSS, Inc., IBM Company, New York, USA) was used for statistical analysis. Data were first tested for normal distribution using Kolmogorow-Smirnow tests. For comparison of the behavioral data between groups, repeated-measures ANOVA was applied with the between-subjects factor GROUP (NC, CXray, IC, AIA) and the within-subjects factor TIME (baseline and days 1, 3, 7, 14, 21, 28, 35 and 42 after induction of AIA). Post hoc *t*-tests were used to describe differences between groups at different time points whenever ANOVA revealed a significant GROUP × TIME interaction. Statistical analysis of cell counts in the DG in the different experimental groups was performed using a one-way analysis of variance (ANOVA) followed by post-hoc *t*-tests (Tamhane-T2). The IC-wt-group was excluded from the one-way ANOVA due its small n-number but compared to the IC group using a *t*-test for unpaired samples. Furthermore, we performed some correlation analyses between the number of BrdU-positive cells and the parameters swelling, mechanical hyperalgesia at the inflamed knee joint and asymmetry in weight bearing using Pearson correlation analysis. P-values <0.05 were considered significant.

## Results

### Assessment of Inflammation, Pain-related and Locomotor Behavior

**Figure 2:**
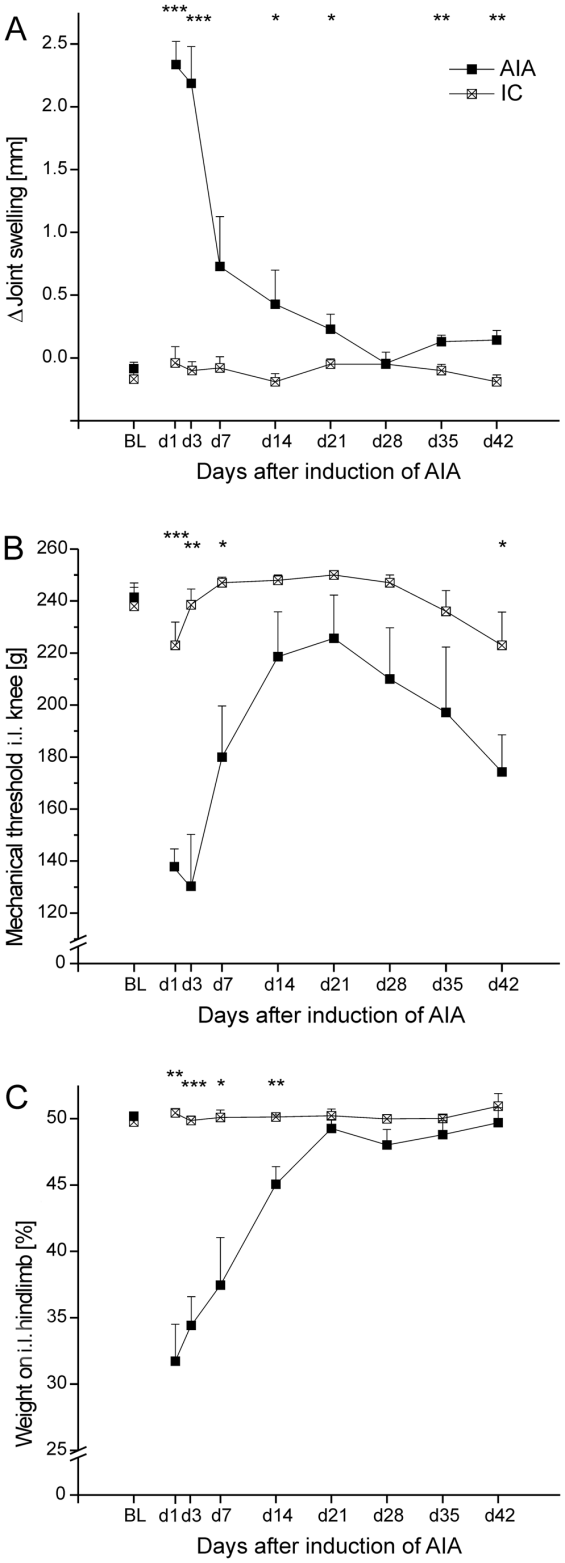
Swelling and pain-related behavior in the AIA and IC groups. (A) Joint swelling displayed as delta (Δ) between ipsilateral (inflamed) and contralateral (noninflamed) knee joints during the observation period of 42 days. (B) Primary mechanical hyperalgesia assessed by measuring the withdrawal threshold to pressure applied to the knee. (C) Weight bearing of the rats shown as the relative body weight resting on the inflamed hindlimb. Data are presented as means ± SEM. *p<0.05; **p<0.01; ***p<0.001. BL - baseline.

The injection of mBSA into the knee joint of immunized rats induced a pronounced unilateral inflammation (AIA), similar as in previous experiments [35]. Whereas immunized control rats (IC) did not exhibited joint swelling, AIA rats showed pronounced swelling particularly at the very acute stage (day 1–3) of AIA (Fig. 2A). The comparison between the IC and AIA groups showed a significant GROUP × TIME interaction [F(8,8) = 10.525; p<0.01]. The AIA-group showed again some swelling of the ipsilateral knee joint in the later chronic stage.

Application of pressure onto the knee evoked a withdrawal response. In immunized rats the withdrawal thresholds remained almost stable. But compared to IC rats the AIA rats showed a significant lowering of withdrawal thresholds (Fig. 2B), with a significant GROUP × TIME interaction [F(8,8) = 17.254; p<0.001]. Thus AIA caused pronounced mechanical hyperalgesia at the inflamed knee, particularly at days 1–3. Interestingly, in the further course of AIA the thresholds decreased again. Weight bearing (Fig. 2C) remained symmetric in IC rats but became asymmetric in AIA rats, as a sign of pain-related guarding behavior (significant GROUP × TIME interaction [F(8,8) = 13.15; p<0.01]).

**Figure 3:**
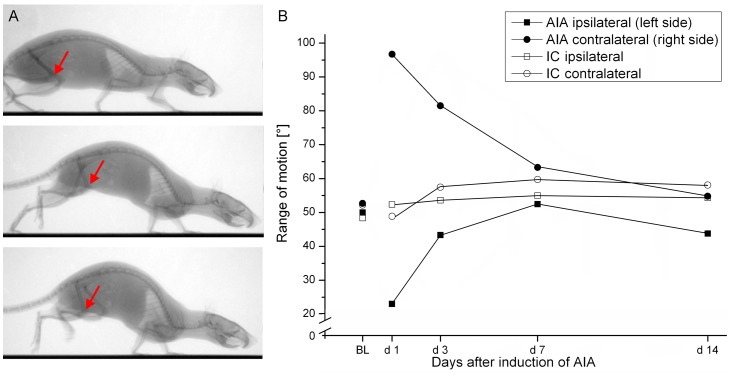
Radiovideographic analysis of locomotion in arthritic and immunized control animals. (A) Walking sequence (top to bottom) of a representative animal of the AIA-group on day 1 after induction of AIA. Arrows show the inflamed knee joint in relieving posture (hardly any change in the joint angle during walking). (B) Range of motion in the inflamed (left side) and non-inflamed (right side) knee joint during the time course of 14 days in a representative animal of the AIA-group (filled symbols) and one animal of the immunized control group (IC, open symbols). BL - baseline.

Using videoradiography, we documented the locomotor behavior (similarly as in a previous comprehensive study in which we analyzed the gait pattern in a large group of rats [36]). We determined the range of motion (ROM) in both knees. Data from representative animals of the AIA and the IC group are shown in Figure 3. ROM in the inflamed knee joint of the AIA rat was strongly reduced but in the contralateral non-inflamed knee joint of the AIA rat ROM increased from about 50° baseline-level to nearly 100° on day 1 and slowly dropped to baseline level on day 14. In IC rats ROM was stable in both knee joints.

### Quantification of BrdU-positive Cells in the Dentate Gyrus (DG)

**Figure 4:**
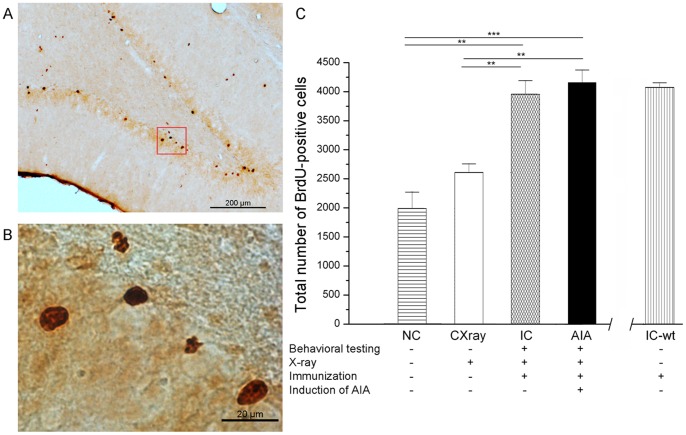
Number of BrdU-positive cells in the DG in different experimental groups (see table under the abscissa;+done, - not done). (A) Overview and (B) magnified detail (red square) of a 40 µm thick coronal section through the DG 42 days after the first BrdU-injection. BrdU-positive cells are labelled dark brown. (C) The NC group (n = 7) and the CXray group (n = 7) were not significantly different, and the AIA group (n = 7) did not differ from the IC group (n = 10). Both the AIA and the IC group had significantly increased numbers of BrdU-positive cells compared to the NC and CXray groups. The column on the right shows another group of 3 rats which were only immunized but not tested for pain-related or locomotor behavior (IC-wt). This group did not significantly differ from the IC group. Data are presented as means ± SEM. **p<0.01; ***p<0.001.

After the in-life phase of 42 days, BrdU-positive cells in the DG were quantified in the 5 experimental groups. Fig. 4A displays a DG section with labelled cells, and Fig. 4B shows a representative part of this section at higher magnification. Labelled cells showed intense dark brown staining which allowed reliable counting. Fig. 4C displays the average numbers of labelled cells in the 5 experimental groups. As there was no significant difference between the ipsi- and the contralateral hemisphere across all subgroups, values are presented as the sum of both dentate gyri. One-way ANOVA revealed an overall significance for the differences between the numbers of BrdU-positive cells (F_(3,27) = _19.858; p<0.001). The control group without any treatment (NC) exhibited 1990±283 cells (n = 7 rats). Seven rats which just underwent irradiation with X-rays (CXray) exhibited on average 2609±151 BrdU-positive cells which is not different from the number of proliferated cells in NC rats (p = 0.414). Rats which were only immunized (IC, n = 10 rats) showed 3957±233 cells, and this number is significantly higher than BrdU-positive cells in either the NC or the CXray group (p<0.01). AIA rats (AIA, n = 7 rats) exhibited 4155±218 BrdU-positive cells which is not different from the number in immunized rats. Thus the main increase of BrdU-positive cells was observed after immunization, and no further increase was found during arthritis.

In addition, 3 rats (IC-wt) were immunized but they were not tested for changes in pain- and locomotor behavior. Thus they can be directly compared to the IC group to assess the influence of behavioral testing alone on the total number of BrdU-positive cells. The column on the right in Figure 4C shows that this group exhibited 4074±81 BrdU-positive cells which is not different from the IC group (p = 0.645), strongly supporting the conclusion that immunization can be regarded as the main driver for neurogenesis in this model.

### Analysis of the Phenotype of BrdU-positive Cells

**Figure 5:**
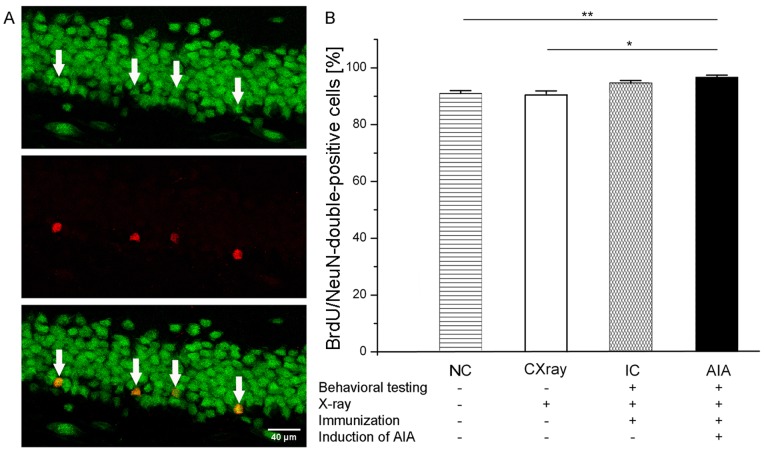
Percentage of BrdU/NeuN-double-positive cells in the DG in different experimental groups (display as in Figure 4). Thirty BrdU-positive cells of each animal were analyzed for coexpression of NeuN. (A) Confocal images of an immunofluorescent-labelled section of the DG. NeuN-labelled cells are green and BrdU-positive cells are red. In the merged picture (lowest one) arrows show BrdU-NeuN-double-positive cells. (B) There were no significant differences between the NC and the CXray group (p* = *1.000) and between the AIA and IC group (p* = *0.476). AIA group showed a significantly increased percentage of mature neurons compared to NC and CXray group. Data are presented as means ± SEM. *p<0.05; **p<0.01.

To identify BrdU-positive cells 42 days after the first BrdU-injection, 30 BrdU-positive cells of the DG were analyzed in each animal for coexpression of the neuronal nuclei marker NeuN. A specimen of immunofluorescent double-staining of cells in the DG is shown in Fig. 5A. NeuN-labelled cells are green (upper panel) and BrdU-positive cells are red (middle panel), and double-labelled cells are in yellow (lower panel). One-way ANOVA revealed an overall significance for the differences between the experimental groups concerning the percentages of BrdU/NeuN-double-positive cells (F_(3,27)_ = 8.339; p<0.001). Almost all BrdU-positive cells in AIA and IC rats were also NeuN-positive (97% in AIA rats and 95% in IC rats), in the NC- and CXray group 91% of BrdU-positive cells were NeuN-positive which is slightly but still significantly less than in the AIA rats. Essentially most BrdU-positive cells were neurons.

### Lack of Correlation between Swelling, Pain and Number of BrdU-positive Cells

**Figure 6:**
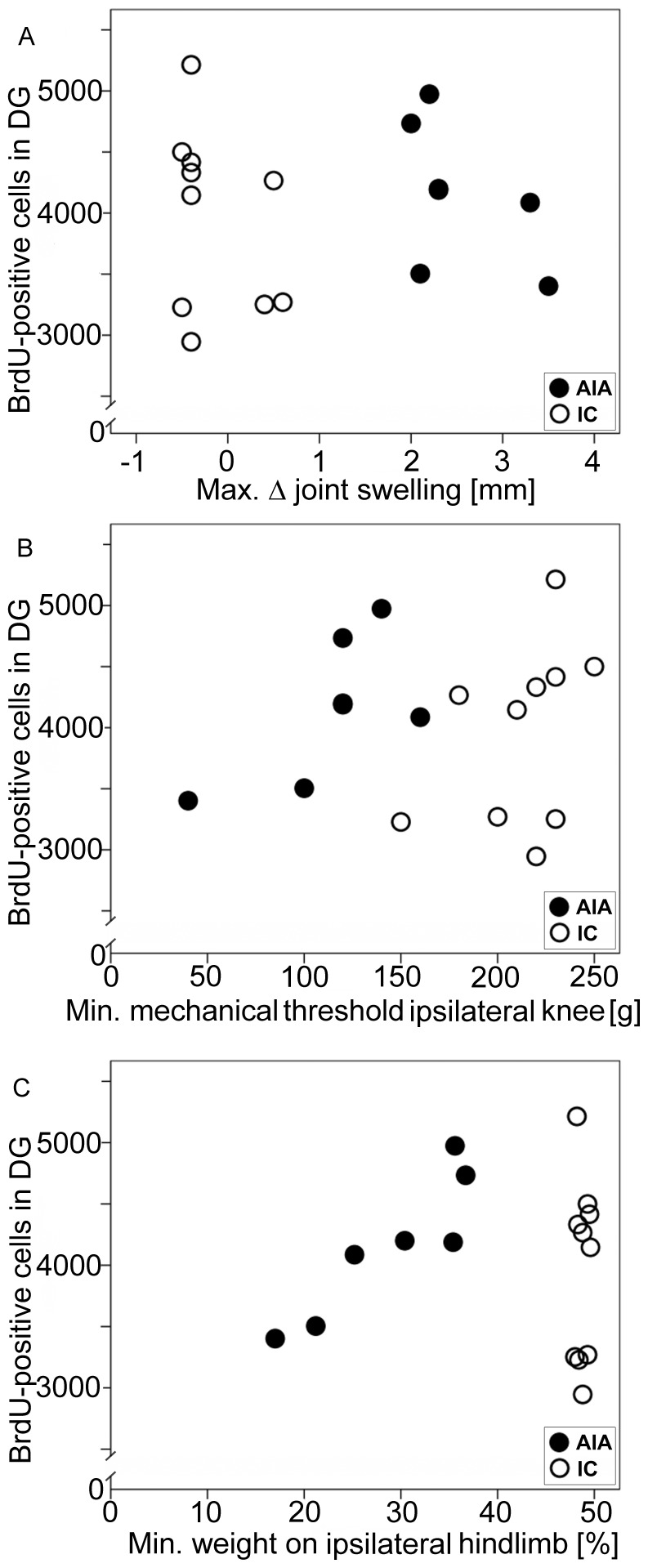
Lack of correlation between swelling or nociceptive behavior and the total number of BrdU-positive cells in the DG in immunized controls (IC) and AIA rats. Each circle and dot shows the maximal swelling or pain behavior of an individual rat during the observation period of 42 days. Circles: animals just immunized (IC group), Dots: animals with AIA. (A) Maximum joint swelling versus total BrdU-positive cells. (B) Maximum primary hyperalgesia (minimum mechanical threshold) at the inflamed knee joint versus total BrdU-positive cells. (C) Maximum asymmetry (least force on the inflamed hindlimb) between left and right hindlimb versus total BrdU-positive cells.

Figures 4 and 5 show that on average AIA animals exhibit the same increase of neurogenesis as immunized but non-arthritic animals. Figure 6 shows an additional analysis of the data taking into account the severity of inflammation and pain and neurogenesis in individual animals. We plotted for each AIA rat the strongest swelling of the inflamed knee joint, the lowest mechanical threshold at the inflamed knee, the lowest weight on the inflamed hind limb during the AIA, and the number of BrdU-positive cells. The same procedure was used for rats which were only immunized. Fig. 6A shows that the distribution of BrdU-positive cells was similar in the immunized group without joint swelling and the AIA group with joint swelling. There was no correlation between swelling and the total number of BrdU-positive neurons (r = 0.009, p = 0.973). Fig. 6B shows the reduction of mechanical threshold at the knee in AIA rats. Again the distribution of BrdU-positive cells was similar in the AIA and IC group, and no correlation was found between mechanical threshold and number of BrdU-positive cells (r = 0.134, p = 0.607). Fig. 6C displays the symmetry of weight bearing in IC animals and the asymmetry in AIA rats, but there was no correlation between the magnitude of asymmetry in weight bearing and the number of BrdU-positive cells (r = 0.071, p = 0.787).

## Discussion

The present study addressed neurogenesis in the dentate gyrus during antigen-induced arthritis in the adult rat. Specifically we investigated whether changes of neurogenesis can be attributed to inflammation and/or to the arthritis-induced neurological symptoms pain and altered locomotion. We found significantly enhanced neurogenesis in rats with unilateral AIA which exhibited significant mechanical hyperalgesia at the inflamed knee joint and pronounced alteration in their locomotor behavior. However, neurogenesis was similarly enhanced in rats which were only immunized but in which no AIA was induced and which showed normal locomotor behavior and no pain. Thus, in the present study enhanced neurogenesis was significantly associated with the immunization process and neither with manifest additional local inflammation alone nor with significant alteration of locomotor behavior and hyperalgesia at the inflamed knee.

A possible confounding factor of the study was the use of X-rays because X-irradiation may reduce the proliferation of neuronal precursor cells from the subgranular zone in the DG [19], [44]–[48]. Mizumatsu et al. [49] found that their lowest dose of X-irradiation tested, namely 2 Gy, caused a significant decrease in the number of proliferating cells relative to sham-irradiated animals in two-month-old male C57BL/J6 mice, and the number of immature neurons in the subgranular zone was strongly reduced as well. In the present experiments radiation alone did not significantly alter neurogenesis, neither in the total number of BrdU-positive cells nor in the percentage of BrdU/NeuN-coexpression compared to non-irradiated controls. The irradiation intensity of the digital high-speed X-ray system used in the present study was 14.3 µSv per second. For x-rays, 1 Gy equates 1 Sv, and therefore 2 Gy equates an irradiation time of 38.85 hours. As animals walk quickly through the irradiation-area we assume an average exposure time to x-rays of at most 2 seconds per animal for all recorded runs on each of the 10 testing days (2 pre-tests for habituation and assessment of baseline levels before the beginning of the experimental time-course of 42 days and 8 test-days during these 42 days), i.e. about 20 seconds total irradiation time over a period of 52 days per animal. This dose does not seem to alter neurogenesis.

Compared to non-immunized controls (NC and CXray) the total number of BrdU-positive cells was more than doubled in both the immunized and the AIA rats, and the percentage of BrdU/NeuN-double-positive cells was also elevated in both groups. Immunized rats and AIA rats both received BrdU injections three weeks after the first immunization, at the time point at which the antigen was injected into the knee in the AIA group. Because AIA develops rapidly within several hours and shows a peak of swelling, hyperalgesia and asymmetry of weight bearing within the first three days of AIA, an effect of local AIA on neurogenesis should be detected by this protocol of BrdU application. However, immunized rats and AIA rats showed similar numbers of BrdU-positive cells. The important conclusion is, therefore, that the development of manifest inflammation did not further stimulate neurogenesis than the previous immunization. The lack of correlation between neurogenesis and swelling (and between neurogenesis and pain-related behavior) supports this conclusion because the numbers of BrdU-positive cells showed a similar distribution in both experimental groups.

A previous study investigated neurogenesis in antigen-induced arthritis in mice [34]. These authors assessed neurogenesis at different time points of AIA, and compared to non-immunized controls they found an increase of proliferating doublecortin- (DCX-) positive cells at day 7 of AIA but neither at day 3 nor at day 21 of AIA. Our results agree with the conclusion that antigen-induced arthritis is associated with enhanced neurogenesis but we believe that neurogenesis in our experiments was rather increased by the immunization than by the arthritis itself. If arthritis would have further increased neurogenesis the numbers of BrdU-positive cells should have been even higher in the AIA group. A ceiling effect of immunization is unlikely because much higher numbers of BrdU-positive cells can be reached under suitable experimental conditions [28], [42], [50].

Interestingly, systemic or intrathecal administration of lipopolysaccharide (LPS) and the ensuing systemic inflammation were shown to be rather detrimental for hippocampal neurogenesis [31], [32]. Wu et al. [33] reported that LPS treatment inhibits the neuronal differentiation pathway but not the proliferation of multipotent and neural stem cells because they found a significant decrease of BrdU/DCX-double-positive cell numbers (DCX is a neuronal progenitor marker) but no alterations in the total number of BrdU-positive cells in the DG after repetitive peritoneal LPS treatment. Thus, immunization for AIA and challenge with LPS apparently exert different effects on neurogenesis.

As a consequence of AIA in the knee, rats exhibited pronounced changes both pain-related and locomotor behavior for more than one week. Derived from previous data that exercise and learning increase the proliferation of neural progenitor cells in the dentate gyrus [15], [51]–[54] we had the hypothesis that neurogenesis may also be enhanced in rats which are using a different locomotor pattern. Wurm et al. [43] observed a significant increase of the number of BrdU-positive cells in the DG of male Wistar rats (age 10 to 12 weeks) that underwent daily skilled forelimb training either after focal cortical infarct or after sham operation (without infarct) but the increase of BrdU-positive cells was less in the infarct group because obviously the infarct itself rather reduces neurogenesis in the DG. However, in the present study immunized rats with normal locomotion exhibited the same increase of BrdU-positive cells as AIA rats with strongly altered locomotion. We have no evidence, therefore, that the pronounced alteration of walking enhanced neurogenesis *per se.* The same applies to the hyperalgesia at the knee joint. The present study does not support a causal relationship between hyperalgesia and enhanced neurogenesis. One could speculate, however, that the immunization-evoked neurogenesis may have facilitated the rapid adaptation of the walking cycle during inflammation, and in this adaptation pain may also be involved.

A previous study reported that repeated injection of complete Freund’s adjuvant (CFA) into the paw induced a reduction of neurogenesis similar as chronic stress evoked by repeated immobilization of animals, and it was concluded that persistent pain produces stress-like alterations (inhibition) in hippocampal neurogenesis [55]. An inhibition of neurogenesis by psychosocial and physical stressors was also reported by other studies [18]–[22]. These data seem to be in contrast to the data of Wolf et al. [34] and of the present study which reported a significant increase of neurogenesis during AIA. However, there are pronounced differences in the CFA model and the AIA model. In the CFA model local inflammation, thermal and mechanical hyperalgesia are generated within 24 hours after injection of CFA whereas in the AIA model the immunization *per se* does not lead to local inflammation and hyperalgesia. These data suggest, therefore, that immunization and overt pain/stress are producing opposite effects on neurogenesis. The lack of a further increase of neurogenesis after elicitation of AIA in the knee and the concomitant appearance of pain is in line with such a concept.

The factor stress has to be taken into account. Rats were repeatedly anesthetized when they received injections for immunization, AIA induction and BrdU application. Furthermore, rats were transported in boxes to the X-ray system, and they were tested for pain-related behavior and the locomotor pattern. However, the CXray group which was transported to the X-ray system was not significantly different from the NC group which was just kept in the cages. Because stress rather decreases neurogenesis (see above), the increase of neurogenesis during immunization is unlikely to be caused by stress.

More likely is a role of certain cytokines which may inhibit or stimulate neurogenesis. Inhibition of neurogenesis was observed for IL-1β, IL-6 or TNF-α all of which are upregulated by LPS [29]–[33], [56], [57]. Natural killer- and T-cell derived IFN-γ may have neurotoxic effects but some studies found that IFN-γ increased neurogenesis [30] and that *in vitro* administration of IFN-γ to NSCs or neuronal cell lines enhanced neuronal differentiation [58]. IFN-γ-transgenic mice exhibited increased proliferation and differentiation of neural stem cells in the adult DG [59]. In the murine AIA model used here IFN-γ was elevated on day 1 of AIA and in the chronic stage whereas IL-6, TNF-α and IL-1β were only transiently enhanced [60].

In summary, this study shows significant stimulation of neurogenesis in the DG associated with AIA. However, the exploration of the factors which are responsible for the increase of neurogenesis provided the unexpected and surprising result that neither manifest inflammation in the joint nor pain and altered locomotion are responsible for the increase of neurogenesis. Only the process of immunization could be causally linked to enhanced neurogenesis. Thus, this study indicates that an immune process may stimulate neurogenesis even if there are no obvious symptoms of a disease. It seems noteworthy to further explore this novel finding in different aspects. The functional consequences of enhanced neurogenesis upon immunization should be explored. It may be speculated that enhanced neurogenesis contributes to the generation of disease defense mechanism. On the other hand, as shown for recovery following stroke, enhanced neurogenesis can also contain elements of maladaptation. Furthermore it seems attractive to elucidate which mechanisms are at work in the interaction of adaptive immune responses and neurogenesis.
